# Inactivation of Src-to-Ezrin Pathway: A Possible Mechanism in the Ouabain-Mediated Inhibition of A549 Cell Migration

**DOI:** 10.1155/2015/537136

**Published:** 2015-03-19

**Authors:** Hye Kyoung Shin, Byung Jun Ryu, Sik-Won Choi, Seong Hwan Kim, Kyunglim Lee

**Affiliations:** ^1^Graduate School of Pharmaceutical Sciences, College of Pharmacy, Ewha Womans University, Seoul 120-750, Republic of Korea; ^2^Laboratory of Translational Therapeutics, Bio-Organic Science Division, Pharmacology Research Center, Korea Research Institute of Chemical Technology, Daejeon 305-600, Republic of Korea

## Abstract

Ouabain, a cardiac glycoside found in plants, is primarily used in the treatment of congestive heart failure and arrhythmia because of its ability to inhibit Na^+^/K^+^-ATPase pump. Recently ouabain has been shown to exert anticancer effects but the underlying mechanism is not clear. Here, we explored the molecular mechanism by which ouabain exerts anticancer effects in human lung adenocarcinoma. Employing proteomic techniques, we found 7 proteins downregulated by ouabain in A549 including p-ezrin, a protein associated with pulmonary cancer metastasis in a dose-dependent manner. In addition, when the relative phosphorylation levels of 39 intracellular proteins were compared between control and ouabain-treated A549 cells, p-Src (Y416) was also found to be downregulated by ouabain. Furthermore, western blot revealed the ouabain-mediated downregulation of p-FAK (Y925), p-paxillin (Y118), p130CAS, and Na^+^/K^+^-ATPase subunits that have been shown to be involved in the migration of cancer cells. The inhibitory effect of ouabain and Src inhibitor PP2 on the migration of A549 cells was confirmed by Boyden chamber assay. Anticancer effects of ouabain in A549 cells appear to be related to its ability to regulate and inactivate Src-to-ezrin signaling, and proteins involved in focal adhesion such as Src, FAK, and p130CAS axis are proposed here.

## 1. Introduction

**Figure 1:**
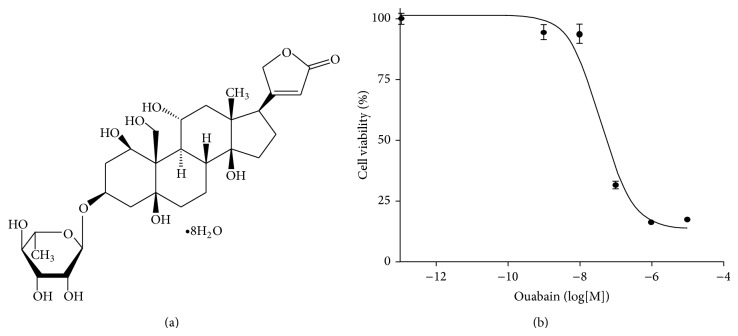
Structure of ouabain and its effect on viability of A549 cells. (a) Structure of ouabain. (b) The effect of ouabain on the viability of A549 cells. Cells (1 × 10^4^ cells/well) in a 96-well plate were incubated with ouabain for 24 h and cell viability was measured by counting.

Ouabain (Figure 1(a)) is a cardiac glycoside found in plants and is primarily used in the treatment of congestive heart failure and cardiac arrhythmia because it inhibits the Na^+^/K^+^-ATPase pump leading to a sequence of events including increase in the level of calcium ions and cardiac contractile force. A recent unexpected epidemiological finding that cancer patients receive cardiac glycosides showed significantly lower mortality rates sparked new interest in possible anticancer properties of cardiac glycosides [1–4]. Prassas and Diamandis [3] confirmed that cardiac glycosides exert antiproliferative and/or apoptotic effects on breast, prostate, lung, renal, pancreatic, melanoma, leukemia, neuroblastoma, and myeloma cancer cells in vitro. But the underlying molecular pathways have not been clarified.

Most of the previous studies of proteomic profile changes resulting from ouabain treatment focused on Na^+^/K^+^-ATPase suppression and were conducted in vascular smooth muscle cells (VSMCs) or in the endothelial cells (ECs) in order to identify the proteins involved in ouabain-induced regulation of cell proliferation and apoptosis and vascular remodeling [5–8] but not the proteins involved in ouabain's anticancer effects.

In this context we conducted a proteomic analysis of human lung adenocarcinoma A549 cells, treated with ouabain to identify the proteins altered when ouabain exhibits its anticancer effects, and thus it is possibly responsible for its anticancer effects.

## 2. Methods

### 2.1. Materials

Ouabain octahydrate and PP2 (Src inhibitor) were purchased from Sigma (MO) and Calbiochem EMD Millipore (Darmstadt, Germany), respectively. Sources of other chemicals and reagents are indicated as they appear in the text.

### 2.2. Cell Culture

Human lung adenocarcinoma A549 cells were cultured in Dulbecco's modified Eagles's medium (DMEM, HyClone, UT) supplemented with 10% fetal bovine serum (FBS, HyClone), 100 U/mL of penicillin, and 100 *μ*g/mL Streptomycin (HyClone) in humidified atmosphere of 5% CO_2_ at 37°C. The culture medium was changed every 3 days.

### 2.3. Cell Viability Assay

Cells in a 96-well plate (1 × 10^4^ cells/well) were treated with ouabain octahydrate for 24 h and cell viability was determined in triplicate by Cell Counting Kit-8 (CCK-8; Dojindo Molecular Technologies, Rockville, MD, USA) according to the manufacturer's protocol.

### 2.4. Sample Preparation and 2-Dimensional Electrophoresis (2-DE)

These were performed as essentially as described by Park et al. [9]. In brief, cells were treated with ouabain for 24 h in DMEM with 10% FBS. The cells were harvested and samples were suspended in 0.5 mL of 50 mM Tris buffer containing 7 M urea, 2 M thiourea, 4% (w/v) CHAPS, and 16 *μ*L protease inhibitor cocktail (Roche Molecular Biochemicals, IN) and sonicated on ice. The sonicates were homogenized and centrifuged at 12,000 ×g for 15 min. Its protein content was quantitated by the Bradford method (Bio-Rad, CA). Fifty units of benzonase (250 units/*μ*L; Sigma, MO) was added to the stock and stored at −80°C until use. For 2-DE analysis, pH 3–10 nonlinear IPG strips (Habersham Biosciences) were rehydrated in a swelling buffer containing 7 M urea, 2 M thiourea, 0.4% (w/v) DTT, and 4% (w/v) CHAPS. The protein lysates (300 *μ*g) were cup-loaded into the rehydrated IPG strips using a Multiphor II apparatus (Amersham Biosciences) set for a total of 57 kVh. The 2D separation was performed on 12% SDS-polyacrylamide gels. Following fixation of the gels for 1 h in 40% (v/v) methanol containing 5% (v/v) phosphoric acid, the gels were stained with colloidal Coomassie Blue G-250 solution (ProteomeTech, South Korea) for 5 h. They were then destained in 1% (v/v) acetic acid for 4 h and imaged using a GS-710 imaging calibrated densitometer (Bio-Rad). Protein spot detection and 2D pattern matchings were carried out using ImageMaster 2D Platinum software (Amersham Biosciences).

### 2.5. In-Gel Digestion of Protein Spots with Trypsin and Extraction of Peptides

The procedure for in-gel digestion of protein spots from Coomassie Blue stained gels was carried out as described in [9]. In brief, protein spots were excised from stained gels and cut into pieces. The gel pieces were washed for 1 h at room temperature in 25 mM ammonium bicarbonate buffer, pH 7.8, containing 50% (v/v) acetonitrile (ACN) and dehydrated in a SpeedVac for 10 min and rehydrated in 10 *μ*L (20 ng/*μ*L) of sequencing grade trypsin solution (Promega, WI). After incubation in 25 mM ammonium bicarbonate buffer, pH 7.8, at 37°C overnight, the tryptic peptides were extracted with 5 *μ*L of 0.5% TFA containing 50% (v/v) ACN for 40 min with mild sonication. The extracted solution was reduced to 1 *μ*L in a vacuum centrifuge. The resulting peptide solution was desalted using a reversed-phase column [10] and subjected to mass spectrometric analysis. A GEloader tip (Eppendorf, Hamburg, Germany) constricted was packed with Poros 20 R2 resin (PerSpective Biosystems, MA). After an equilibration with 10 *μ*L of 5% (v/v) formic acid, the peptide solution was loaded on the column and washed with 10 *μ*L of 5% (v/v) formic acid. The bound peptides were eluted with 1 *μ*L of *α*-cyano-4-hydroxycinnamic acid (CHCA) (5 mg/mL in 50% (v/v) ACN/5% (v/v) formic acid) and dropped onto a MALDI plate (96 × 2; Applied Biosystems, CA).

### 2.6. Analysis of Peptides Using MALDI-TOF MS and Identification of Proteins

The masses of the tryptic peptides were determined with a Voyager-DE STR mass spectrometer (PerSpective Biosystems) in reflectron positive ion mode as described in [11]. External calibration was performed for every four samples with mixtures of adrenocorticotropic fragment 18–39 (monoisotopic mass, 2465.1989), neurotensin (monoisotopic mass, 1672.9175), and angiotensin I (monoisotopic mass, 1296.6853) as standard calibrants, and mass spectra were acquired for the mass range of 900–3500 Da. The proteins were identified through matches in Swiss-Prot and NCBI databases, using the search program ProFound (http://prowl.rockefeller.edu/prowl-cgi/profound.exe), MASCOT (http://www.matrixscience.com/cgi/search_form.pl?FORMVER=2&SEARCH=PMF), or MS-Fit (http://prospector.ucsf.edu/prospector/cgi-bin/msform.cgi?form=msfitstandard, University of California San Francisco, Version 4.0.5). The following mass search parameters were set: peptide mass tolerance, 50 ppm; a mass window between 0 and 100 kDa, allowance of missed cleavage, 2; consideration for variable modifications such as oxidation of methionine and propionamides of cysteines. Only significant hits as defined by each program were considered initially with at least 4 matching peptide masses.

### 2.7. Western Blotting

Cells (2 × 10^5^ cells/well) placed in a 6-well plate were treated with ouabain octahydrate for the indicated time, washed twice with cold PBS, and scraped into a lysis buffer containing 50 mM Tris-HCl (pH 7.4) 150 mM NaCl, 1 mM EDTA, 2 mM Na_3_VO_4_, 1 mM NaF, 0.25% deoxycholate, 1% Triton X-100, and a protease inhibitor cocktail tablet (Roche, Diagnostics, Mannheim, Germany). The cell lysate was centrifuged at 15,000 ×g for 10 min at 4°C. The protein level of the supernatant was determined using the BCA protein assay kit (Pierce, Rockford, IL, USA). Samples (20 *μ*g) were mixed with sample buffer (100 mM Tris-HCl, 2% sodium dodecyl sulfate, 1% 2-mercaptoethanol, 2% glycerol, and 0.01% bromophenol blue) and incubated at 95°C for 10 min. To detect the Na^+^/K^+^-ATPase *α*1 and *β*1, samples were incubated at 75°C for 15 min. Samples were subjected to sodium dodecyl sulfate-polyacrylamide gel electrophoresis. Equivalent amounts of the proteins separated on gels were transferred onto nitrocellulose membranes (Whattman, Germany) and stained with Ponceau S to confirm efficiency of transfer. Membranes were washed, blocked with TBST (10 mM Tris-HCl, pH 7.5, 150 mM NaCl, and 0.1% Tween 20) containing 3% BSA, for 1 h at room temperature, and probed with primary antibody overnight at 4°C. They were washed three times with TBST for 30 min, incubated with secondary antibody conjugated to horseradish peroxidase (Santa Cruz Biotechnology, Inc., TX) for 2 h, and washed three times with TBST for 30 min. Antibodies against p-ezrin (Y353), ezrin, p-Src (Y416), Src, p-paxillin (Y118), paxillin, p-FAK (Y925), and FAK, respectively, were purchased from Cell Signal Technology (MA). Antibodies against Na^+^/K^+^-ATPase *α*1 and *β*1 were purchased from Upstate, Merck Millipore (MA), and antibodies against Na^+^/K^+^-ATPase *α*2 and *α*3 were purchased from Santa Cruz Biotechnology and Thermo Fisher Scientific (MA), respectively. Antibodies against p130CAS and actin were purchased from R&D System and Santa Cruz Biotechnology, Inc., respectively. Membranes were developed with Amersham ECL Plus (GE Healthcare Bio-sciences, Sweden) using the LAS-3000 luminescent image analyzer (Fuji Photo Film Co., Ltd., Japan).

### 2.8. Phosphokinase Antibody Array Analysis

Phosphokinase array analysis was performed using Proteome Profiler Human Phosphokinase Array Kit (R&D systems) according to the manufacturer's procedure. Briefly, cells (5 × 10^5^ cells) in a 60 mm^2^ dish were treated with ouabain octahydrate for 24 h, washed twice with cold PBS, lysed with the lysis buffer 6 in the kit, and centrifuged at 14,000 ×g for 5 min. The protein levels of the supernatants were assayed using BCA kit (Pierce, Rockford, IL, USA). For Human Phosphokinase Array assay, preblocked nitrocellulose membranes were incubated with 300 *μ*g of cellular extracts overnight at 4°C on a rocking platform. The membranes were washed three times with 1x wash buffer in the kit to remove unbound proteins and then incubated with a mixture of biotinylated antibodies and streptavidin-HRP antibodies. Amersham ECL Plus was applied to determine spot densities. Array images were analyzed using Multi Gauge 3.0 (Fuji Photo Film Co., Ltd., Japan).

### 2.9. Cell Migration Assay

Cell migration assay was performed as described previously with some modification [12]. A549 cells (5 × 10^5^ cells) were treated with various concentrations of ouabain (1, 10, and 100 nM) for 4 h in a 60 mm culture dish and harvested with trypsin EDTA. After recounting the harvested cells, their immigrations were assayed in a Boyden chamber (Neuro Probe, MD, USA) as follows. The lower compartment of a 48-well Boyden chamber was filled with 30 *μ*L of DMEM containing 0.1 and 10% FBS. An 8.0 *μ*m pore polycarbonate membrane (Neuro Probe) was coated with gelatin solution (0.01% gelatin and 0.1% acetic acid in distilled water) for 24 h and a 50 *μ*L volume of a cell (2 × 10^4^ cells) suspension was introduced into the upper compartment of the chamber, and the chamber was incubated at 37°C for 6 h. The membrane was fixed and stained with Diff-Quik solution (Dade Behring, DE, USA) and placed on a microscope slide. The invading cells were counted using a light microscope at 100x magnification. The data are presented as means ± standard deviation of 4 fields from each well of triplicate samples. Statistical significance was determined using Student's* t*-test and differences of *P* < 0.05 were considered significant.

## 3. Results

### 3.1. Ouabain Decreased the Viability of A549 Cells in a Dose Dependent Manner and Changes the Expression of some Cellular Proteins

**Figure 2:**
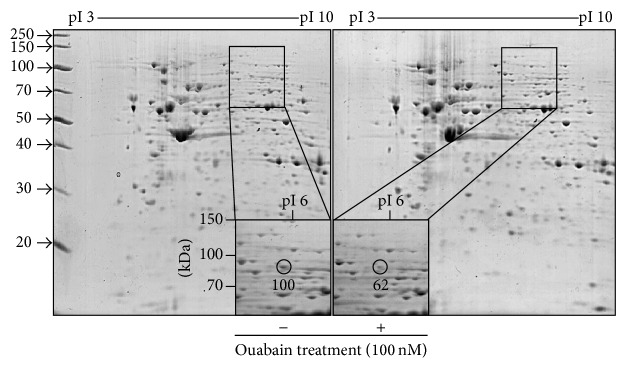
2-DE analysis. The relative volume of circle-indicated spot was analyzed by ImageMaster 2D Platinum software. MALDI-TOF-MS spectrum of the circled peptide spot after in-gel digestion.

The effect of ouabain on the viability of A549 cells was assessed by counting viable cells (Figure 1(b)) and changes in protein expression in the cells were assessed using two-dimensional (2D) gel electrophoresis. IC_50_ of ouabain on the viability of A549 cells was about 40 nM (Figure 1(b)). In order to identify the proteins that might be involved in the anticancer activity of ouabain, we performed a comparative proteomic analysis of lysates of control A549 cells and cells treated with 100 nM ouabain. Of over 500 protein spots that appeared in the 2-DE gel, two spots showed increases in proteins and 7 spots showed decreases in proteins (Figure S1 and Table S1 in the Supporting Information available online at http://dx.doi.org/10.1155/2014/537136). The circled spot in Figure 2 shows a 62% decrease in ouabain-treated A549 cells compared to control. This circled spot was digested in gel with trypsin and subjected to peptide mass fingerprinting (PMF).

### 3.2. Ouabain Decreased the Expression of Ezrin

**Figure 3:**
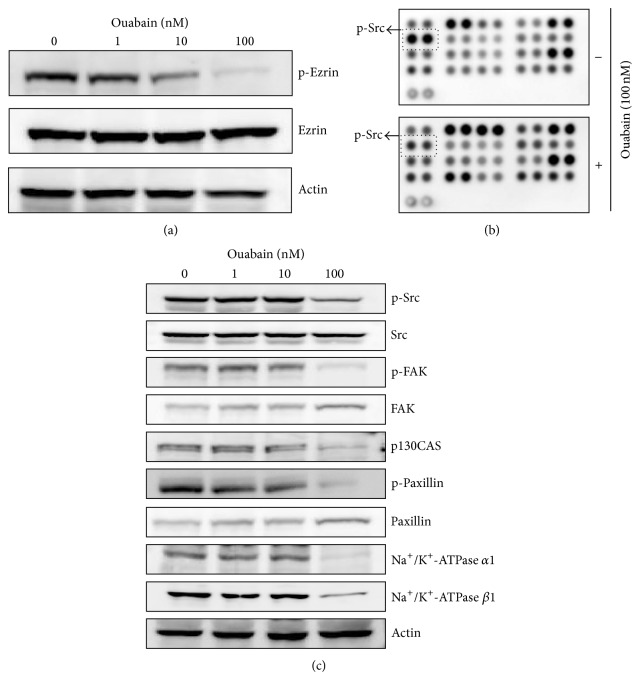
Proteome profiler array analysis of phosphokinase and validation. (a) Effects of ouabain on the expression and phosphorylation of ezrin were evaluated by Western blot analysis. Actin was used as an internal control. (b) For phosphokinase array study, 300 *μ*g of proteins obtained from A549 cells (5 × 10^5^ cells in a 60 mm^2^ dish) treated with vehicle (DMSO) or ouabain octahydrate for 24 h in the membranes was probed. (c) Effects of ouabain on the expression and/or phosphorylation of Src, FAK, p130CAS, paxillin, and Na^+^/K^+^-ATPase subunits were evaluated by Western blot analysis.

Based on the PMF, the estimated pI and molecular weight by 2-DE map, the circle-indicated protein in the 2-DE gel was identified as ezrin. These characteristics are listed in Table 1. Ouabain-induced decrease in the ezrin signal in 2-DE gel was further differentiated by Western blot analysis. As shown in Figure 3(a), ouabain dose-dependently decreased the level of phosphoezrin (Y353), but not that of total ezrin (Figure 3(a)). Further, we carried out phosphokinase array analysis to investigate molecular pathways that potentially contribute to ouabain-mediated cell death. We found that p-Src (Y416) was downregulated by ouabain in 39 intracellular proteins in the control and ouabain-treated A549 cells (Figure 3(b)). Ouabain-mediated decrease of p-Src (Y416) was also confirmed by Western blot analysis (Figure 3(c)).

Since ezrin protein family and Src are known to play roles in membrane-cytoskeleton interactions and focal adhesion, respectively, we further investigated the effect of ouabain on the expression and activation levels of other molecules related to focal adhesion in A549 cells by Western blot analysis. These included focal adhesion kinase (FAK) and cytoskeletal proteins such as p130CAS and paxillin. Figure 3(c) shows that ouabain decreased the phosphorylation of FAK (Y925) and paxillin (Y118) and the expression of p130CAS.

### 3.3. Ouabain Inhibited Src-Mediated Cell Migration

**Figure 4:**
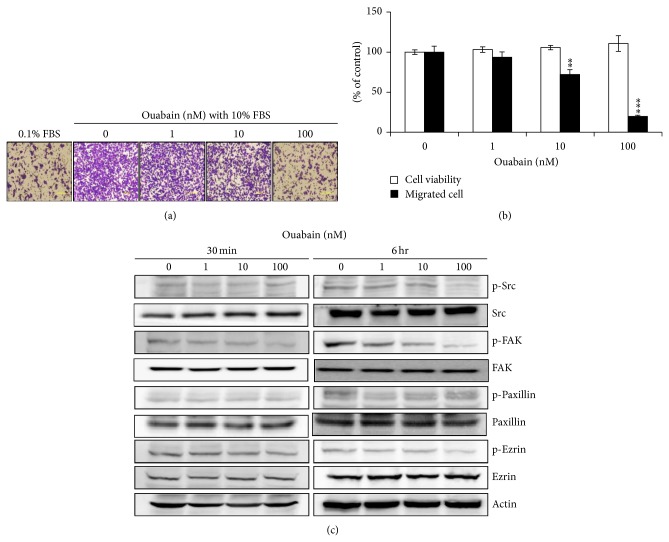
Effect of ouabain on migration of A549 cells. (a) In vitro migration assay was performed twice in triplicate using a 48-well Boyden chamber with a gelatin-coated polycarbonate membrane. DMEM containing either 0.1% FBS or 10% FBS was added into the bottom chamber and cells were loaded into the upper chamber and incubated at 37°C for 6 h. The cells on the upper side of the membrane were removed, and the cells on the bottom of the filter membrane were stained with Diff-Quick solution. (b) The numbers of migrated cells were counted under a light microscope. The data are presented as mean ± standard deviation (^*^ *P* < 0.05; ^**^ *P* < 0.01; ^***^ *P* < 0.001).

Human A549 cells have been reported to be highly metastatic, and cardiac glycosides have been reported to inhibit the migration of cancer cells by the specific inhibition of the Na^+^/K^+^-ATPase *α*1 subunit [13, 14]. Therefore, we examined the effect of ouabain on the expression of Na^+^/K^+^-ATPase subunits and the migration of A549 cells using the Boyden chamber analysis. As shown in Figure 3(c), ouabain treatment for 1 day of A549 cells strongly inhibited the expression levels of Na^+^/K^+^-ATPase *α*1 and *β*1. Furthermore, it decreased the migration of A549 cells in a dose-dependent manner even when it was exposed to A549 cells for 4 h before migration (Figures 4(a) and 4(b)).

Binding of cardiac glycosides to Na^+^/K^+^-ATPase is known to activate several downstream signaling pathways, including phospholipase C (PLC), mitogen-activated protein kinase (MAPK), phosphatidyl-inositol-3-kinase (PI3 K), and Src kinase [15–17]. To determine whether ouabain exerts its antimigration effect by inactivating ezrin and paxillin via Src inhibition, the phosphorylation levels of signaling molecules including Src, FAK, paxillin, and ezrin were assessed by Western blotting. As shown in Figure 4(c), ouabain decreased the phosphorylated level of FAK in 30 min and its decrease was maintained up to 6 hr. The phosphorylated levels of Src and ezrin, but not of paxillin, were shown to be decreased by ouabain in 6 hr.

**Figure 5:**
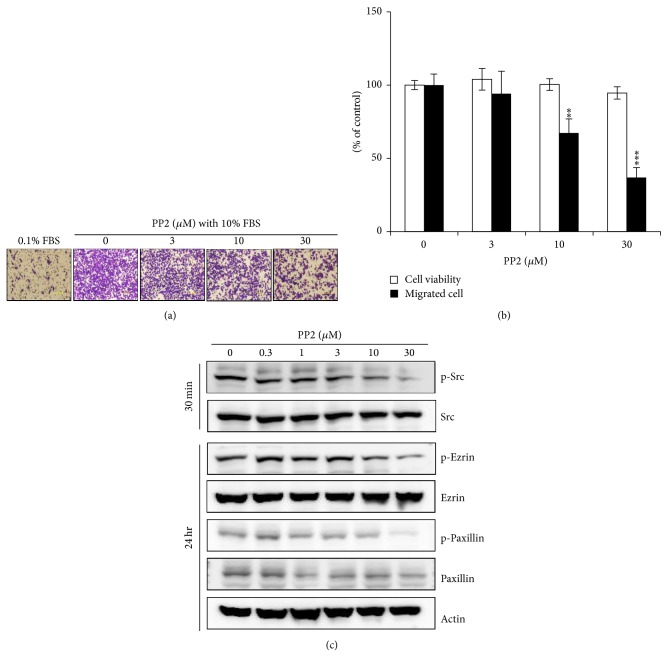
Effects of Src inhibitor, PP2, on cell migration and on the phosphorylation of ezrin and paxillin. (a) In vitro migration assay was performed twice in triplicate using a 48-well Boyden chamber with a gelatin-coated polycarbonate membrane. Serially diluted PP2 was added into the bottom chamber and cells were loaded into the upper chamber. Following incubation at 37°C for 6 h, the cells on the upper side of the membrane were removed, and the cells on the bottom of the filter membrane were stained with Diff-Quick solution. (b) The numbers of migrated cells were counted under a light microscope. The data are presented as mean ± standard deviation (^*^ *P* < 0.05; ^**^ *P* < 0.01; ^***^ *P* < 0.001). (c) Src inhibitor, PP2, was treated for indicated time and then Western blot analysis was performed as described in Materials and Methods.

The involvement of Src in the antimigration activity of ouabain was further confirmed by the pharmacologic inhibition study. Src inhibitor, PP2, also exhibited significant inhibition effect in the migration of A549 cells from the top to the bottom chamber at 6 h, in a dose-dependent fashion when several doses of PP2 (3, 10, and 30 *μ*M) were administered in the bottom chamber (Figures 5(a) and 5(b)). Additionally, A549 cells were treated with PP2 for 30 min and after 24 h the phosphorylation of signaling molecules including Src, ezrin, and paxillin was assessed by Western blotting. As shown in Figure 5(c), PP2-induced inhibition of Src resulted in reduced phosphorylation of ezrin and paxillin. These results suggest that ouabain exerts its antimigration effect by inactivating ezrin and paxillin via Src inhibition.

## 4. Discussion

Anticancer effect of ouabain has been reported in several cancer cells including A549 cells [18]. This study confirmed that ouabain exerts strong antiproliferative activity on A549 cells at nanomolar concentrations (IC_50_, 40 nM). Also our results are in agreement with previous reports that ouabain* per se* significantly inhibits the growth of A549 cells by inducing cell arrest, but not by apoptosis at nanomolar concentrations which correlate with the inhibition of Na^+^/K^+^-ATPase [18, 19]. Furthermore, the finding that ouabain-induced inhibition of tumor growth accentuates irradiation damage led to the suggestion that ouabain may have clinical application in radiotherapy [20].

In this study we employed proteomics technology to identify proteins that change in A549 cells in response to ouabain and possibly inhibit the cancer cells. Recently, starvation-induced autophagy has been suggested to account for the growth inhibitory effect of ouabain in A549 [18]. In our study, A549 cells were treated with ouabain in the presence of FBS and eliminated the possibility that we may be identifying proteins regulated by cell culture conditions including starvation-induced autophagy. Among 9 proteins regulated by ouabain in A549, p-ezrin was confirmed to be regulated by ouabain in a dose-dependent manner.

Ezrin is a member of the cytoskeleton-associated protein family and is involved in a wide variety of cellular processes [21]. Importantly, ezrin has been reported to play an important role in the metastasis of lung cancers [22, 23]. The expression and clinical significance of ezrin in lung cancers have been related to phosphoezrin protein expression in tumor tissues found to be higher in precancerous tissues and in benign pneumonic tissues [24]. Levels of phosphoezrin were found to correlate with the invasiveness of tumors in several types of cancers [25]. Phosphoezrin (Y353) required for transmitting a survival signal during epithelial differentiation has been suggested to be a potent prognosis predictor for pancreatic cancer [26, 27]. In this study, we found that ouabain dose-dependently decreased the level of phospho-ezrin (Y353).

Phosphorylation of ezrin at Y353 was found to be mediated through Src tyrosine kinase in prostate cancer [28], and it is a crucial element of Src-induced features in malignant cells [29]. The phosphorylation of Src at Y416 in the activation loop of the kinase domain upregulates the enzymatic activity of Src. Interestingly, exposure of human breast MDA-MB-435s cells to 100 nM ouabain caused rapid and transient activation of Src kinase in 5 min (but not in 15 min) and increased the coimmunoprecipitations of Src and Na^+^/K^+^-ATPase *α* subunit with epidermal growth factor receptor when ouabain was incubated for 5 min [20]. However, in this study, we found ouabain-mediated decreases of p-Src (Y416) and Na^+^/K^+^-ATPase subunits in A549 cells after 1-day incubation. These results suggest that ouabain transiently induces the activation of Src kinase and its binding to Na^+^/K^+^-ATPase, but, after this transient activation, both Src and Na^+^/K^+^-ATPase subunits are downregulated by long-term exposure of ouabain.

Ezrin deficiency in highly metastatic human lung carcinoma 95D cells caused the reduction of the cell migration and invasion [30], and Src-FAK signaling is known to regulate the migration of cancer cells. Furthermore, ouabain has recently been shown to inhibit migration of A549 and human lung cancer H292 cells, via suppressing FAK signaling [31, 32], and the involvement of Na^+^/K^+^-ATPase in the migration of cancer cells has been also reported [13, 14]. These results suggest a possible mechanism that involves ouabain-mediated inhibition of A549 cell migration via inactivation of Src-to-ezrin signaling axis.

In many tumor cells, Src forms a complex with FAK to generate signals leading to tumor growth and metastasis [30, 33]. Within this complex, Src transphosphorylates FAK within C-terminal domain (Y925) and provides a binding site for the Grb2/SH2 domain and triggers a Ras-dependent activation of MAP kinase pathway [31]. In Src-transformed cells, Ras signal transduction pathway may be constitutively activated by FAK Y925 phosphorylation. FAK is overexpressed in a variety of cancers and its overexpression in lung cancer leads to the cancer migration and invasion [32]. Additionally, the overall survival was better in FAK-negative than in FAK-positive patients with lung adenocarcinoma [30]. In this study, when A549 cells were incubated with ouabain for 30 min, p-FAK (Y925), but not p-Src, was downregulated. Furthermore, the downregulation of p-Src and p-FAK observed after 6 hr was maintained up to 24 hr.

FAK-Src complex binds to and phosphorylates various adaptor proteins such as paxillin. Here, we found that ouabain decreased the phosphorylation of paxillin (Y118), which has been shown to occur at Y118 [31]. Phosphopaxillin (Y118) can provide a docking site for recruitment of other signaling molecules to focal adhesions [34] and contribute to the control of Src-induced anchorage-independent growth by FAK and adhesion [35].

We also found that another cytoskeletal protein, p130CAS, was also downregulated by ouabain in A549 cells. p130CAS is also a substrate of FAK and plays an important role in regulating focal adhesion, driving cell migration. Recently, the overexpression of p130CAS has been observed in 61.9% of lung cancers suggesting that p130CAS may impact a variety of clinicopathological features of lung cancer and may influence the prognosis of lung cancer patients [23].

Our ongoing studies include the investigation on the ouabain-induced signaling such as Src, ezrin, FAK, and p130CAS in other lung carcinoma cells including H460 cells. To dissect the mechanism of ERM proteins in the signaling described herein, effects of local phosphorylation of ERM proteins by ouabain are worthy of investigation using the immunofluorescence experiments or phosphospecific antibodies. Also, it was reported, recently, that small molecule inhibitors of ezrin inhibit lung metastasis of ezrin-sensitive cells as well as invasive phenotype of osteosarcoma cells suggesting that novel targeted therapy that directly or indirectly inhibits the function of ezrin might be a rational approach to prevent tumor metastasis [36]. In this regard, studies on the inhibitory effects of an ezrin inhibitor, NSC668394, on the ezrin-related signaling such as Src and paxillin and on the migration of cancer cells are underway in our researches.

## 5. Conclusions

We showed here that the anticancer effects of ouabain in A549 cells are related to its ability to regulate ezrin, Na^+^/K^+^-ATPase subunits, and proteins involved in the signaling of focal adhesion such as Src, FAK, and p130CAS. Furthermore, we also proposed that a possible mechanism for ouabain-mediated inhibition of A549 cell migration is inactivation of Src-to-ezrin signaling axis. The present study suggests that molecules involved in Src-to-ezrin signaling axis offer a new target for lung cancer therapy.

## Supplementary Material

Supplementary Figure S1: Differentially expressed proteins by ouabain treatment in A549 cells. Circled spots represented the differentially expressed proteins that were further identified by MALDI-TOF MS.
Supplementary Table S1: MALDI-TOF-based identification.

## Figures and Tables

**Table 1:** MALDI-TOF-based identification of ezrin.

Protein name	NCBI BLAST	Number of matched peptides	Sequence coverage (%)	Theoretical Mr(Da)/pI	Score	Expect
Ezrin	gi∣46249758	21	30	69199/5.94	116	6.1*e* − 07
