# Supplementary material for: Inactivation of Src-to-Ezrin Pathway: A Possible Mechanism in the Ouabain-Mediated Inhibition of A549 Cell Migration
Source: Biomed Res Int. 2015 Mar 19;2015:537136. doi: 10.1155/2015/537136 (PMC4383155; doi:10.1155/2015/537136)
Supplement: Supplementary file 1 — Supplementary Figure S1: Differentially expressed proteins by ouabain treatment in A549 cells. Circled spots represented the differentially expressed proteins that were further identified by MALDI-TOF MS. Supplementary Table S1: MALDI-TOF-based identification. [file 537136.f1.pdf]

Figure 1

A

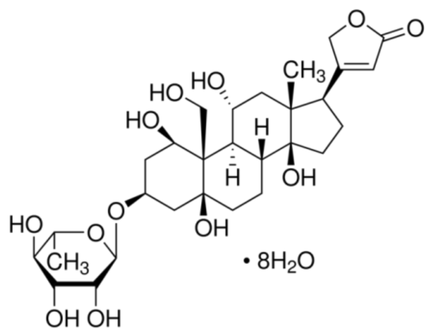

O
O
CH3
HO
HO
HO
H
H
OH
O
OH
HO
O
CH3
OH OH
• 8H2O

B

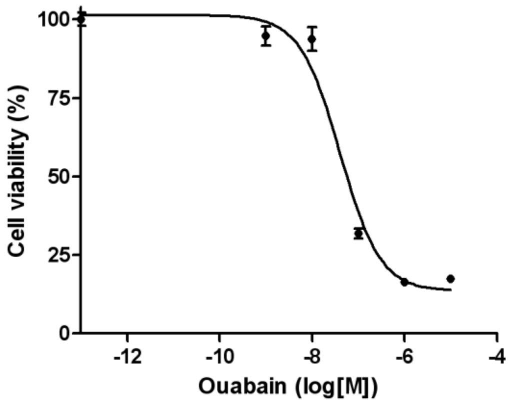

Cell viability (%)
100
75
50
25
0
-12
-10
-8
-6
-4
Ouabain (log[M])

Figure 2

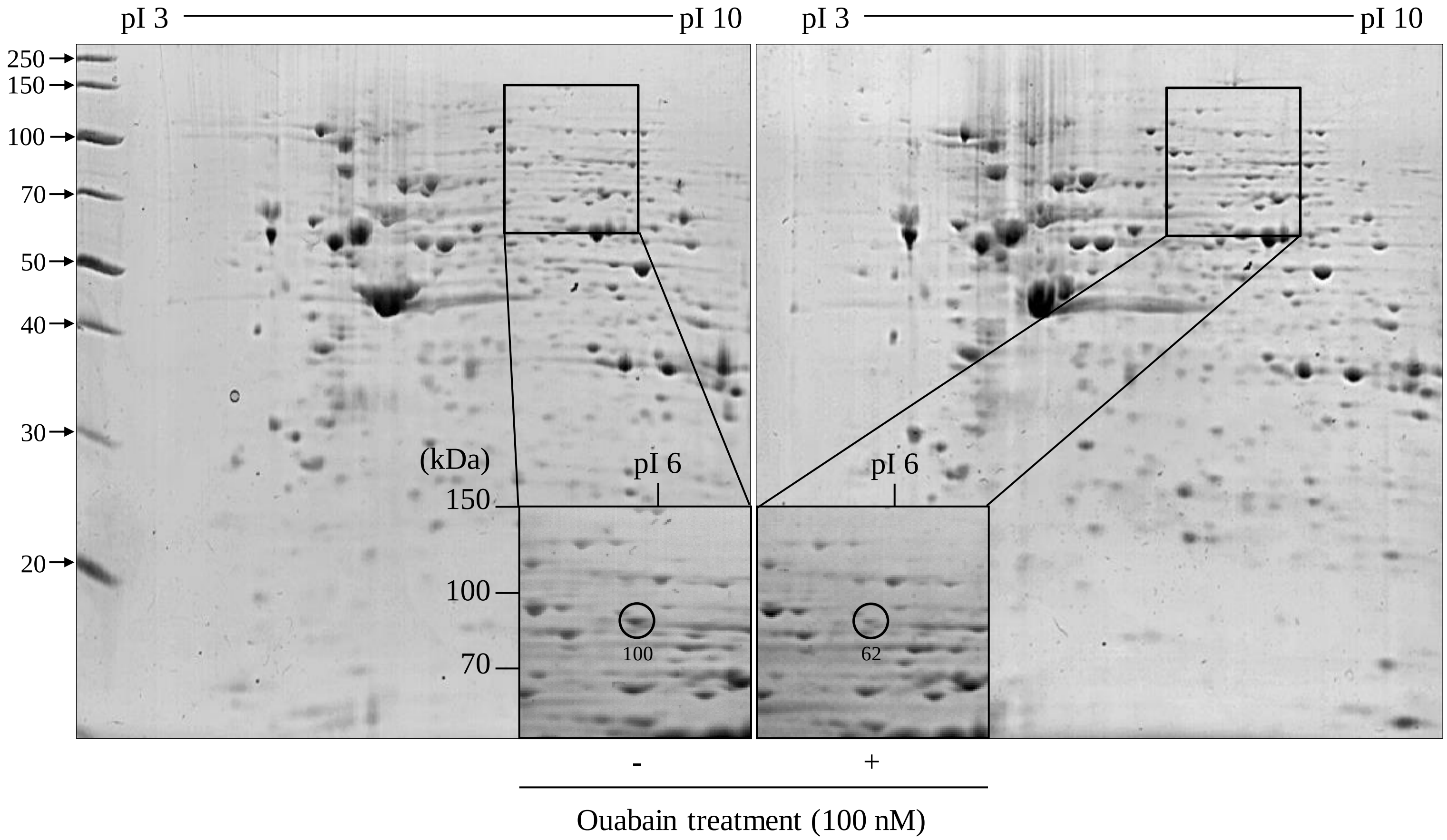

pI 3
pI 10
pI 3
pI 10
250
150
100
70
50
40
30
20
(kDa)
pI 6
pI 6
150
100
70
100
62
-
+
Ouabain treatment (100 nM)

Figure 3

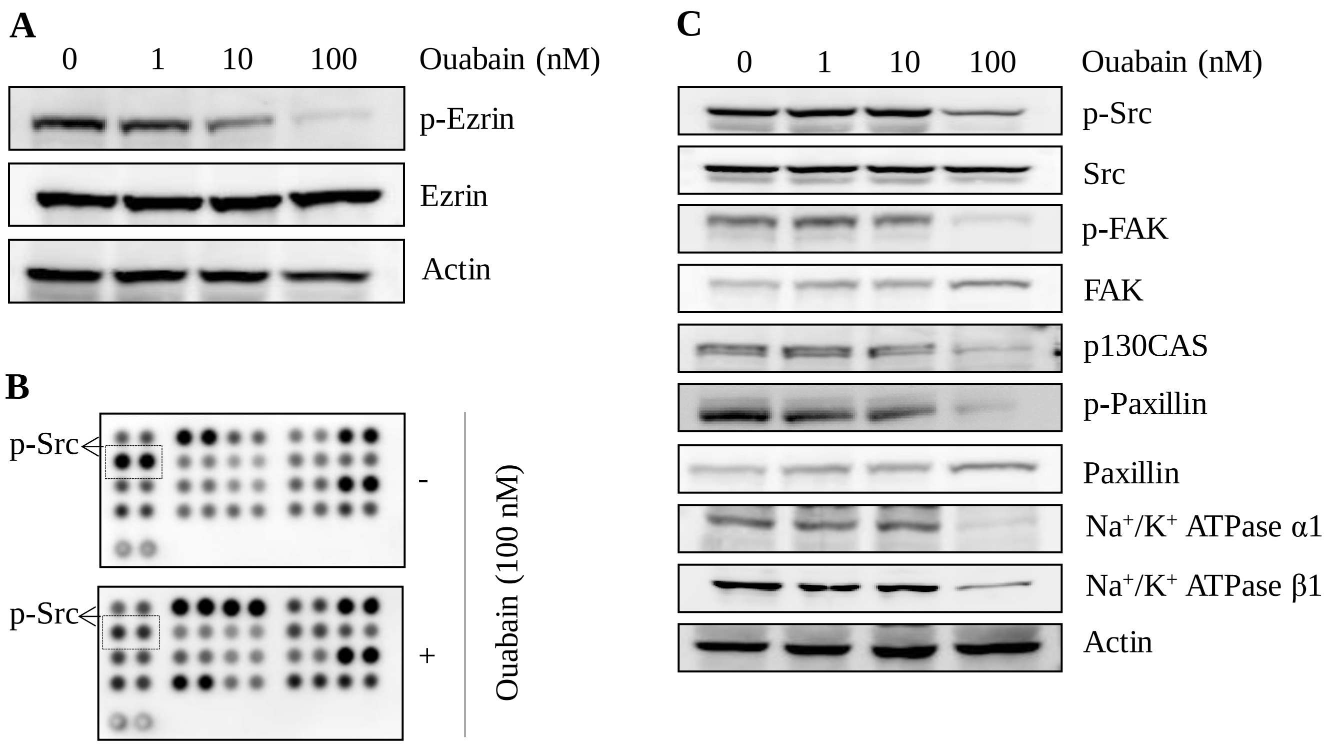

A
0
1
10
100
Ouabain (nM)
p-Ezrin
Ezrin
Actin
B
p-Src
p-Src
-
+
Ouabain (100 nM)
C
0
1
10
100
Ouabain (nM)
p-Src
Src
p-FAK
FAK
p130CAS
p-Paxillin
Paxillin
Na+/K+ ATPase α1
Na+/K+ ATPase β1
Actin

Figure 4

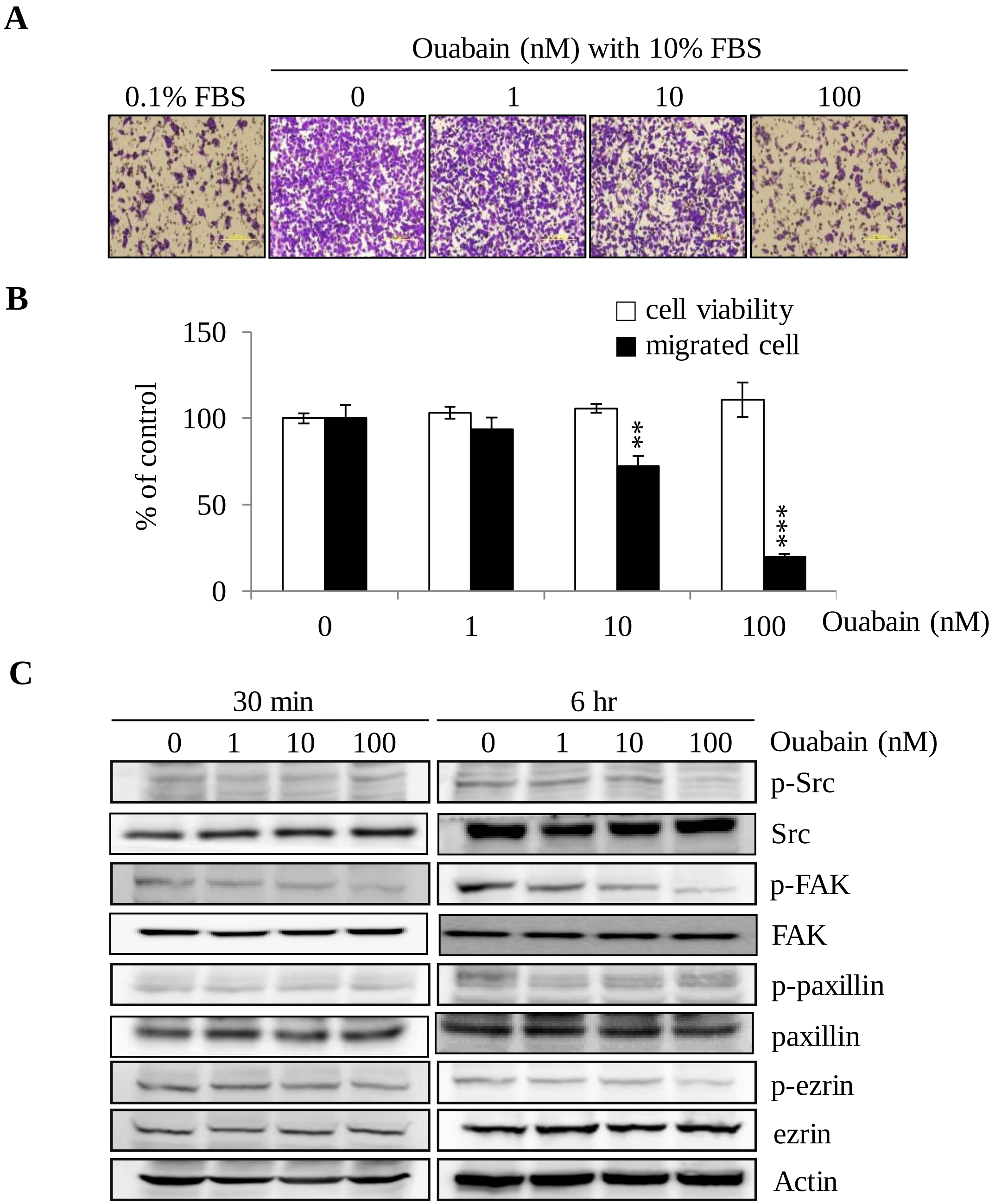

A
Ouabain (nM) with 10% FBS
0.1% FBS
0
1
10
100
B
cell viability
migrated cell
% of control
150
100
50
0
**
***
0
1
10
100
Ouabain (nM)
C
30 min
6 hr
0
1
10
100
0
1
10
100
Ouabain (nM)
p-Src
Src
p-FAK
FAK
p-paxillin
paxillin
p-ezrin
ezrin
Actin

Figure 5

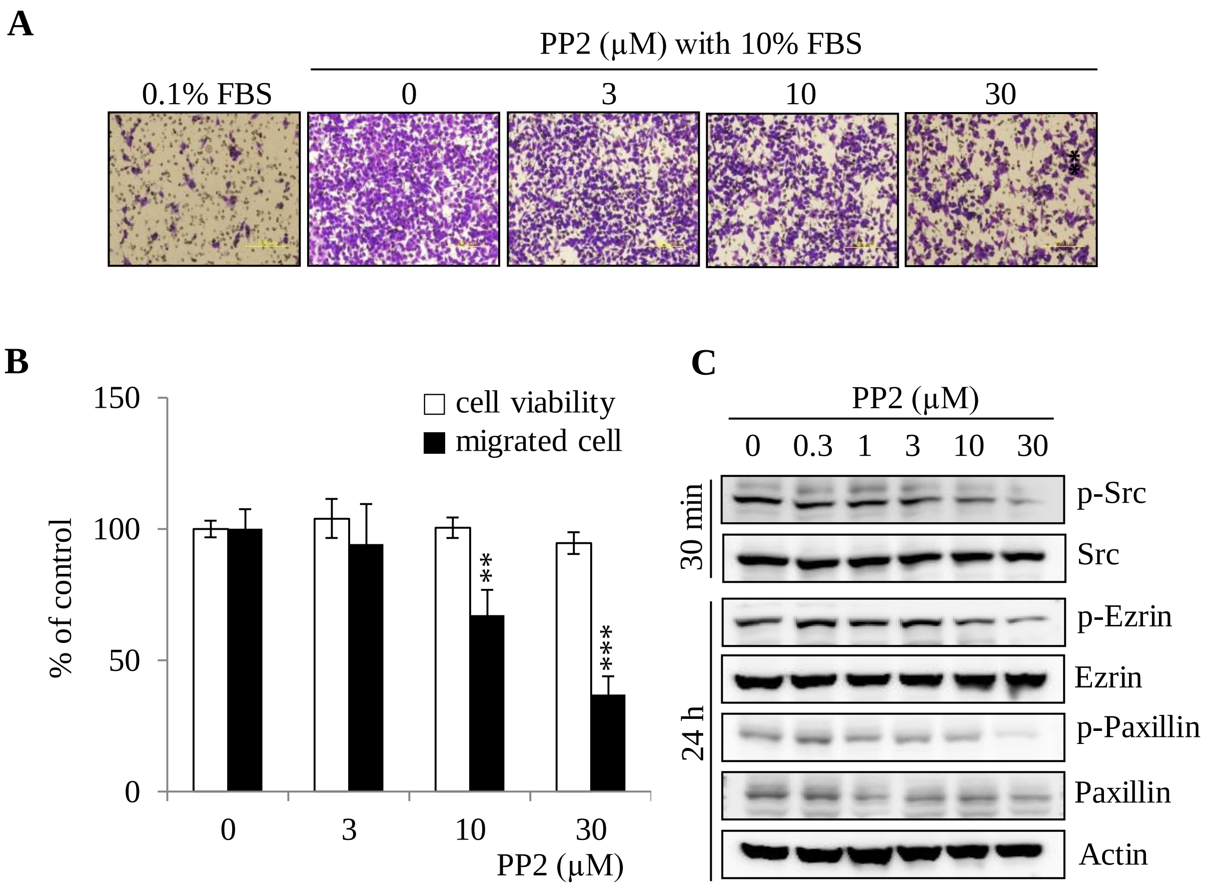
A
PP2 (μM) with 10% FBS
0.1% FBS
0
3
10
30
B
150
100
50
0
% of control
cell viability
migrated cell
**
***
0
3
10
30
PP2 (μM)
C
PP2 (μM)
0
0.3
1
3
10
30
30 min
24 h
p-Src
Src
p-Ezrin
Ezrin
p-Paxillin
Paxillin
Actin

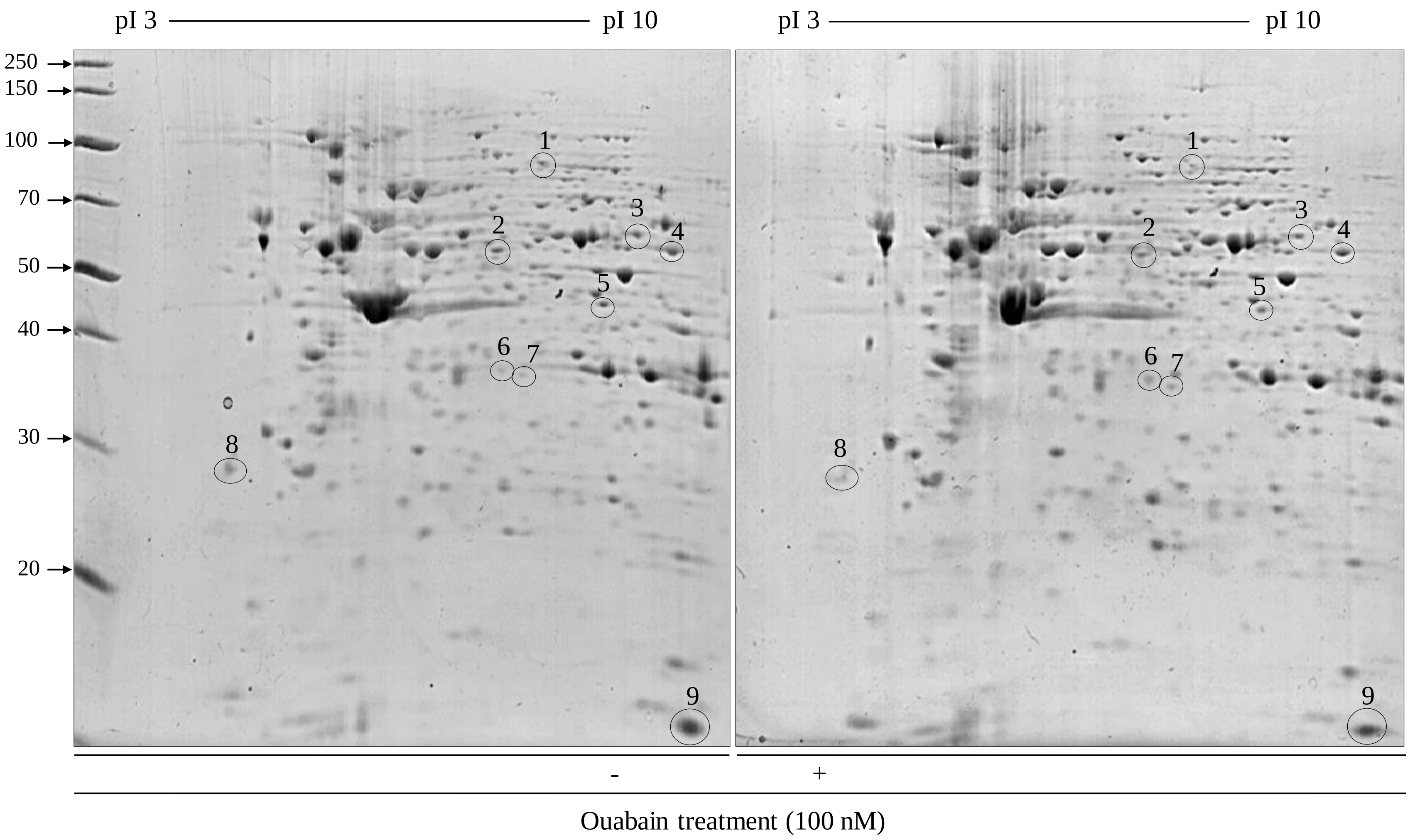

Figure S1. Differentially expressed proteins by ouabain treatment in A549 cells. Circled spots represented the differentially expressed proteins that were further identified by MALDI-TOF MS.
