# Supplementary material for: Inactivation of Src-to-Ezrin Pathway: A Possible Mechanism in the Ouabain-Mediated Inhibition of A549 Cell Migration
Source: Biomed Res Int. 2015 Mar 19;2015:537136. doi: 10.1155/2015/537136 (PMC4383155; doi:10.1155/2015/537136)
Supplement: Supplementary file 2 [file 537136.f2.pdf]

Table S1. MALDI-TOF-based identification

No.	Protein name	NCBI BLAST	No.of matched peptides	Sequence coverage (%)	Theoretical Mr(Da)/pI	Score	Expect	Expression by ouabain
1	Ezrin	gi|46249758	21	30	69.2/5.94	116	6.1e-07	Decreased
2	Chain A, Crystal Structure Of The Aminoacylation Cataytic Fragment Of Human Tryptophanyl-Trna Synthetase	gi|50513261	14	43	43.3/7.12	146	6.1e-10	Decreased
3	HNRNPL protein	gi|211828181	17	21	61.8/7.07	81	0.0018	Decreased
4	Chain A, Structure Of Human Muscle Pyruvate Kinase (Pkm2)	gi|67464392	17	40	59.7/8.22	191	1.9e-14	Decreased
5	CXorf22 protein	gi|20380780	30	24	85.4/8.85	64	0.037	Decreased
6	Ribosomal protein, large, P0	gi|12654583	17	53	34.2/5.42	123	1.2e-07	Increased
7	26S proteasome non-ATPase regulatory subunit 14	gi|5031981	20	47	34.5/6.06	84	0.001	Increased
8	Chain A, Human 3alpha-Hsd Type 3 In Ternary Complex With Nadp And Testosterone	gi|21465695	12	48	36.6/6.86	131	1.9e-08	Decreased
9	Chain A, X-ray Structure Of Nm23 Human Nucleoside Diphosphate Kinase B Complexed With Gdp At 2 Angstroms Resolution	gi|1421609	30	59	17.1/8.55	96	5.40e-05	Decreased
